# Evaluating the Influence of Elevated Temperature on Compressive Strength of Date-Palm-Fiber-Reinforced Concrete Using Response Surface Methodology

**DOI:** 10.3390/ma15228129

**Published:** 2022-11-16

**Authors:** Musa Adamu, Yasser E. Ibrahim, Hani Alanazi

**Affiliations:** 1 Engineering Management Department, College of Engineering, Prince Sultan University, Riyadh 11586, Saudi Arabia; 2 Department of Civil Engineering, Bayero University Kano, Kano P.M.B 3011, Nigeria; 3 Department of Civil and Environmental Engineering, College of Engineering, Majmaah University, Al-Majmaah 11952, Saudi Arabia

**Keywords:** natural fiber, date palm fiber, silica fume, elevated temperature, weight loss, residual compressive strength

## Abstract

Due to its availability and affordable processing, date palm fiber (DPF) is among the natural and sustainable fibers used in cementitious composites. Furthermore, DPF is an agricultural, organic, and fibrous material that when subjected to higher temperature can easily degrade and cause reduction in strength. Therefore, the influence of elevated temperatures on the unit weight and strengths of DPF-reinforced concrete needs to be examined. Under this investigation, DPF is used in proportions of 0–3% weight of binder to produce a DPF-reinforced concrete. Silica fume was utilized as a supplemental cementitious material (SCM) in various amounts of 0%, 5%, 10%, and 15% by weight to enhance the heat resistance of the DPF-reinforced concrete. The concrete was then heated to various elevated temperatures for an hour at 200 °C, 400 °C, 600 °C, and 800 °C. After being exposed to high temperatures, the weight loss and the compressive and relative strengths were examined. The weight loss of DPF-reinforced concrete escalated with increments in temperature and DPF content. The compressive and relative strengths of the concrete improved when heated up to 400 °C, irrespective of the DPF and silica fume contents. The heat resistance of the concrete was enhanced with the replacement of up to 10% cement with silica fume when heated to a temperature up to 400 °C, where there were enhancements in compressive and relative strengths. However, at 800 °C, silica fume caused a significant decline in strength. The developed models for predicting the weight loss and the compressive and relative strengths of the DPF-reinforced concrete under high temperature using RSM have a very high degree of correlation and predictability. The models were said to have an average error of less than 6% when validated experimentally. The optimum DPF-reinforced concrete mix under high temperature was achieved by adding 1% DPF by weight of binder materials, replacing 12.14% of the cement using silica fume, and subjecting the concrete to a temperature of 317 °C. The optimization result has a very high desirability of 91.3%.

## 1. Introduction

In recent years, concrete usage has increased in areas of high temperature applications. When concrete is subjected to elevated temperatures, serious damage might occur because of the induced chemical and physical changes that can cause a significant decline in its mechanical properties and its durability performance. This can further lead to a reduction in the structural integrity of the concrete member, resulting in spalling and, consequently, failure of the concrete structural element [1,2]. The physical deterioration processes brought forward by elevated temperatures have a considerable impact on the longevity of concrete structures, and this can result in detrimental structural breakdowns. Therefore, it is necessary to use caution while choosing suitable materials to limit the negative influences of elevated temperatures on concrete. The performance of concrete whenever it is subjected to an elevated temperature is significantly influenced by the characteristics of the constituent materials used in its production. Some of these characteristics include the density, voids, and thermal conductivity of the aggregates, and the thermal compatibility and adhesion between the cement paste and the aggregate [1,3,4]. When the temperature rises above 110 °C, significant changes in the calcium silicate hydrate (C-S-H), including dehydration and the release of chemically bonded water, occur. Concrete shrinkage also happens as a result of Ca (OH)_2_ dissociation, which occurs at temperatures of 530 °C and above. At 300 °C, dehydration and thermal expansion of the aggregates cause internal stresses that lead to microcracks in the microstructure of concrete [1,4,5]. The Ca(OH)_2_ and C-S-H decomposition takes place at around 400 °C and above 600 °C, respectively. The decomposition of these two hydration products accelerates at temperature around 800 °C, which results in a significant decrease in the concrete’s strength. Furthermore, the conversion of Ca(OH)_2_ to quicklime (CaO) takes place during its decomposition. The reaction is reversible, as the Ca(OH)_2_ can be reformed during the cooling process when the CaO comes into contact with moisture. Series of this repeated reaction cause expansion and contraction and consequently cracking in the concrete’s microstructure [2,6]. One of the ways to decrease the effect of the conversion of Ca(OH)_2_ into lime in concrete under high temperature is by the use of pozzolanic materials, which include silica fume, fly ash and slag, etc., because of their high silica (SiO_2_) content. The SiO_2_ will react with and consume the Ca(OH)_2_ to produce excess products of hydration like calcium aluminate silicate hydrates and C-S-H, thereby reducing the amount of Ca(OH)_2_ in the cement paste [6].

Behnood and Ziari [7] varied the water-to-cement (w/c) ratio in high strength concrete containing silica fume as SCM in proportions of 0%, 6%, and 12% by weight. They subjected the concrete to high temperatures of 20 °C, 100 °C, 200 °C, 300 °C, and 600 °C for 3 h. Their findings demonstrated that the compressive strengths rose for mixtures with 6% and 10% silica fume by 18.06% and 22.92% at 100 °C, 15.58% and 20.1% at 200 °C, 15.31% and 18.16% at 300 °C, and 11.63% and 7.62% at 600 °C, respectively. The high pozzolanic reaction, which resulted in Ca(OH)_2_ consumption and the formation of a dense microstructure, was the main cause of the increase in strength with the silica fume addition. Poon and Azhar [8] investigated the impact of increased temperature on high-strength concrete’s compressive strength. They used silica fume in place of cement in amounts of 0%, 5%, 10%, and 20%. They heated the concrete at 20 °C, 200 °C, 400 °C, 600 °C, and 800 °C. They reported that replacement with the silica fume addition improved the strength of the concrete when heated to 400 °C or higher. At 400 °C, mixes with 5% and 10% silica fume increased in strength by 21.1% and 27.98%, respectively. However, for 600 °C and 800 °C, only a 5% silica fume addition boosted the strength, while a 10% silica fume addition slightly decreased the strength. Meanwhile, Rutkowska, Paweł Ogrodnik [9] reported that fly ash as an SCM in concrete has no effect on the compressive strength of the concrete at high temperatures between 300 °C and 700 °C.

Fibers have been used as additives in concrete to enhance its performance, especially under tensile load. Another benefit of fiber in concrete is it improves its heat resistance by delaying and preventing the thermal crack propagation and spalling of the concrete whenever it is exposed to an elevated temperature [2,10]. Some studies investigated the effect of high temperatures on the strength of fiber-reinforced concrete containing silica fume. Gencel and Nodehi [11] reported that replacing cement with 0–15% silica fume led to the retention of higher compressive strength at up to 800 °C in basalt-fiber-reinforced foam concrete, when they recorded a strength increment of by up to 129% in concrete with silica fume compared to concrete without it. They attributed this increase in strength to the silica fume’s greater thermal resistance when contrasted with cement. Tanyildizi [12] also reported silica fume caused a reduction in the compressive and flexural strengths of polypropylene (PP) fiber when subjected to temperature of 400 °C and above.

DPF is among the most widely available natural fibers, particularly in North Africa and the Middle East countries, obtained mostly as a waste material from palm trees. DPF has been utilized as a composite of cements such as mortar, concrete, and bricks owing to its merits, such as its eco-friendliness, availability, environmental sustainability, low processing cost, and higher strength-to-cost ratio [13,14,15]. The material’s thermal insulation has been discovered to be improved and its thermal conductivity has been decreased via the use of DPF [16,17,18,19]. DPF also enhanced the acoustic characteristics of buildings and composites [17]. Nevertheless, an addition of DPF to cementitious composites has been reported to decrease its mechanical properties and durability performance [14,19,20,21]. To minimize the negative influence of DPF on the mechanical characteristics of composites, supplementary cementitious materials like silica fume could be added because of their great pozzolanic reactivity; thereby, they are expected to densify the concrete microstructure and enhance the bond between the cement matrix and the DPF, hence improving strength. Ibrahim and Adamu [14] demonstrated improved mechanical characteristics by utilizing silica fume for SCM in DPF-reinforced concrete. Silica fume was also employed as SCM in basalt-fiber-reinforced concrete by Gencel and Nodehi [11], as well as Saradar and Nemati [22], who discovered a noticeable improvement in mechanical characteristics.

DPF is a vegetable fiber and an organic material containing high amounts of lignin, hemicellulose, and cellulose, with other organic and inorganic constituents [13,15,23]. Therefore, DPF composites are susceptible to deterioration when subjected to weathering, acidic or alkaline environments, microbes, moisture, etc. [24]. When DPF composites are exposed to severe conditions like high temperature, the DPF is expected to degrade and disintegrate because of its organic vegetable nature. Hence, there is need to study the effect of high temperature on the properties of DPF-reinforced concrete and devise some means of improving its performance under high temperature. On the other hand, silica fume, when used as a supplementary cementitious material, has been reported to improve the heat resistance of both concrete and fiber-reinforced concrete. Additionally, there are few available studies on the effect of temperature on the properties of DPF-reinforced concrete. Most of the available studies focused mainly on the effects of DPF on concrete and mortar under normal environmental conditions, even though, as discussed, high temperature has a significant effect on DPF. Therefore, in this study, silica fume was used as a partial replacement for cement to improve the heat resistance of DPF-reinforced concrete. Furthermore, response surface methodology (RSM) has been used to develop models for predicting the weight loss and relative compressive strengths of the DPF-reinforced concrete under elevated temperatures using DPF and silica fume as variables.

## 2. Materials and Methods

### 2.1. Materials

**Figure 1:**
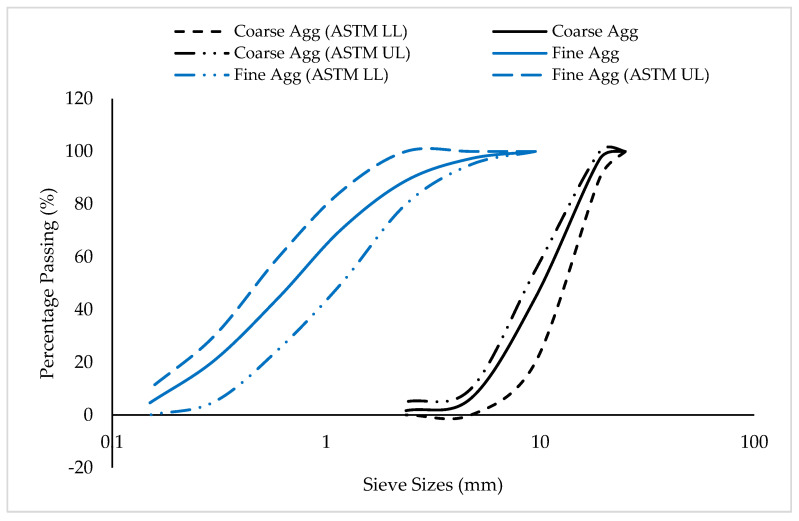
Particle-size distribution of aggregates.

The main binder employed in this study was type I ordinary Portland cement, which met the requirements specified in ASTM C150/C150M [25] and has the chemical properties as given in Table 1. As an SCM, silica fume was used. The specific gravity, specific surface area, and bulk density of the silica fume are 2.25, 18,000 m^2^/kg, and 630 kg/m^3^, respectively. Table 1 also presents the chemical properties of the silica fume. Natural sand that was free of dust and other contaminants and complied with the requirements set out in ASTM C33/C33M [26] was utilized as the fine aggregate. The aggregate has a specific gravity equal to 2.63, a bulk density of 1560 kg/m^3^, a fineness modulus equal to 2.3, and a water absorption rate equal to 1.9%. It was in a saturated, surface-dried condition. The fine aggregate’s sieve examination revealed that it was a well-graded sand with all of its particles falling inside the permitted range specified by ASTM C33/C33M [26]. Figure 1 displays the fine aggregate particle size analysis. As a coarse aggregate, 19 mm maximum-size well-graded gravel was employed. The coarse aggregate, as shown in Figure 1, was properly graded and had a specific gravity of 2.67, a water absorption rate of 0.7%, and a bulk density of 1460 kg/m^3^. The amount of mixing water was decreased by 10%, while a superplasticizer dosage of 1% by weight of binder material was added. The superplasticizer had a density of 1060 kg/m^3^ and was based on polycarboxylate.

The DPF was obtained in its raw state, in a rectangular-form woven mesh of about 300–500 mm in length and 200–300 mm in width. The raw fiber was first separated into single fibers by hand, and then immersed in water and further separated and cut into single-fiber diameters of 0.2–1.0 mm and lengths ranging between 20–30 mm. A 3% NaOH alkaline solution was then prepared, and the single DPF was further immersed in the solution for 3 h to remove dirt, dust, and any other impurities. After that, the fiber was taken out of the alkaline medium and properly cleaned using water. After 48 h of air drying, the fiber was applied to the concrete. Table 2 also lists the DPF’s characteristics.

### 2.2. Mix Proportioning

The mix design and proportioning of the control concrete was done based on the standard specifications outlined in ACI 211.1R [27], adopting the absolute volume method. Different amounts of DPF—0%, 1%, 2%, and 3%—by weight of binder were added to the mixture. By using the volume replacement method, silica fume was employed to partly replace the cement in varying percentages of 0%, 5%, 10%, and 15%. To mitigate the impact of fluctuations in the water-to-cementitious-materials ratio on the properties of the concrete, the superplasticizer dosage was maintained at 1% by weight of cementitious materials across all of the mixes. With the different combinations of DPF and silica fume, 13 mixes were prepared and tested in the laboratory as presented in Table 3. Based on the proportion of variables, i.e., DPF and silica fume, each mix was given a unique identification. For instance, mix D1-0 was the mix with 1% DPF and 0% silica fume, D1-5 contains 1% DPF and 5% silica fume, and D3-15 has 3% DPF and 15% silica fume.

### 2.3. Sample Preparations and Casting

The concrete was sampled, mixed, and cured in accordance to the methods outlined in ASTM C192/C192M [28]. The dried constituent materials were first batched and weighed based on the quantities calculated from the mix design using the batching-by-weight method. Before mixing, the aggregates were brought to dried saturated surface conditions, while the cement, silica fume, and DPF were completely dried and free from any agglomeration. The rotating-drum mixer type was used for mixing the fresh concrete. It was also ensured that the mixer was cleaned and completely dried prior to mixing. The superplasticizer and water were mixed in a container and kept until needed. First, the fine aggregate was poured inside the mixer; the cement and silica fume followed and afterward were mixed for 60 s. The DPF was then combined with the coarse aggregates, and the mixing process was restarted while using the remaining half of the water. As the mixing was still ongoing, the remaining half of the water plus superplasticizer was added continuously. When a homogeneous mix was obtained, the mixing was ended, and the fresh concrete was cast right away into the prescribed molds. The molds had been cleaned, tightened, and oiled before casting the fresh concrete. The casting was carried out in three layers; after each layer was poured into the mold, compaction was carried out using tamping rods, and each layer was tamped 35 times. Upon completing the casting, the surfaces of the samples were leveled and finished. Before demolding, the freshly cast samples were allowed to settle and solidify in the laboratory for around 24 h. The samples of hardened concrete were then given the necessary curing time in clean water before testing. For each of the mixes and each temperature variation, three samples were prepared and tested, and the mean value reported.

### 2.4. Experimental Methods

**Figure 2:**
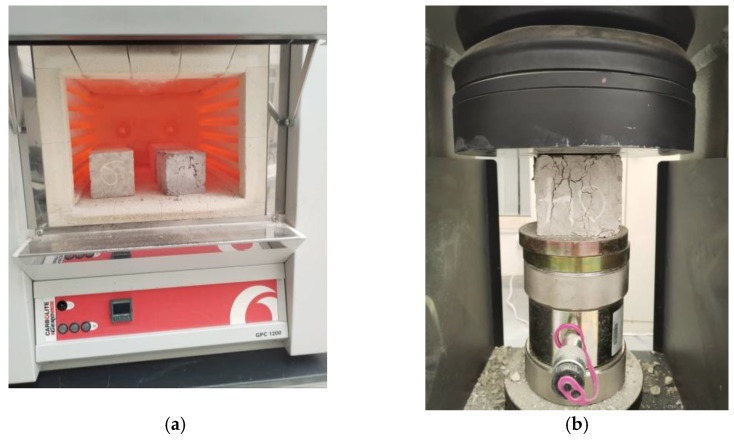
Experimental testing. (**a**) Heating samples furnace at 800 °C; (**b**) compressive strength testing after heating.

Following the 28-day curing period, the concrete cubes (100 mm in size) were removed and dried. Upon drying, the samples were then weighed and then placed in the furnace and subjected to different temperatures of 200 °C, 400 °C, 600 °C, and 800 °C. The samples were heated at the rate of 10 °C/min until they reached the desired temperature. The samples were then heated at the constant desired temperature for 2 h. The heating process of the samples in the furnace is shown in Figure 2a. After 2 h of heating, the furnace was switched off and the samples were allowed to cool completely in the furnace without opening it. After the samples were completely cooled, they were weighed again prior to compressive strength testing. Using a 2000 kN capacity universal testing machine (UTM), the compressive strength test was performed on heated 100 mm cubes in line with the BS EN 12390-3 [29] requirements, as shown in Figure 2b. Three cube samples were tested and the average result reported for each of the mixes. The weight loss for each sample after subjection to elevated temperature was calculated using Equation (1). The relative compressive strength was estimated using Equation (2).

(1) $$W_{L}\left(\%\right)=\frac{W_{n}-W_{t}}{W_{i}}\times 100$$

(2) $$R_{c}=100-\left[\left(\frac{F_{n}-F_{t}}{F_{n}}\right)\times 100\right]$$

where *W_L_* and *R_c_* are the weight loss and relative compressive strength, respectively, in %, *W_i_* is the initial weight of sample before subjecting to elevated temperature in g, *W_t_* is the final weight of the sample after heating and cooling in g. *F_n_* is the compressive strength of the sample at normal temperature in MPa, and *F_t_* is the compressive strength of the sample after temperature *t* under consideration in MPa.

## 3. Results and Discussions

### 3.1. Weight Loss

**Figure 3:**
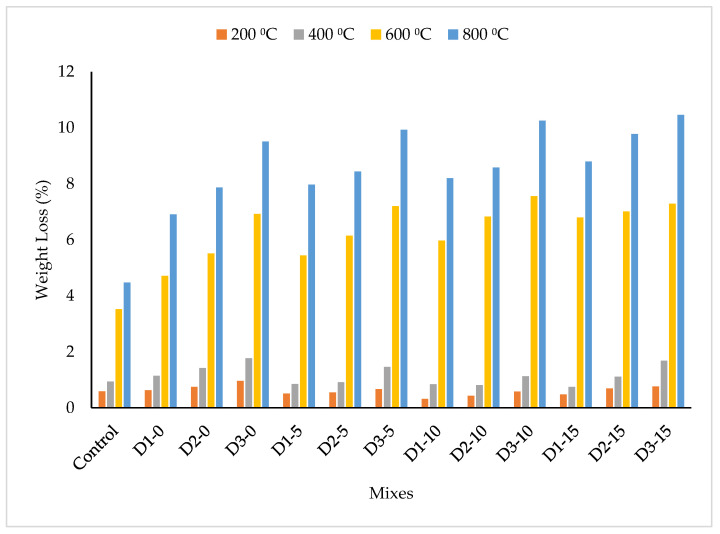
Weight loss caused by elevated temperatures.

Weight loss is one of the properties that is normally calculated to measure the overall physical changes that occur in concrete and mortar when subjected to factors that cause deterioration, such as elevated temperature [11]. The findings on the weight loss of DPF-reinforced concrete containing silica fume when subjected to high temperatures ranging from 200 °C to 800 °C are presented in Figure 3. The weight loss for all the mixes increased with increments of elevated temperature, and at higher temperatures above 400 °C there is a significant escalation of mass loss. Initially, the mass loss was due to the evaporation of gel and capillary water from the concrete’s microstructure, followed by the evaporation of interlayer and absorbed water. As reported by Ramachandran and Feldman [30], as well as Gupta and Siddique [31], the aforementioned loss of water from the concrete matrix occurs between 65–80 °C. Therefore, the increase in weight loss can be attributed to the continual dehydration or release of water from the cement matrix, which led to the emergence of voids, hence reducing the unit weight [1]. Additionally, the increment in temperature led to the deterioration of the concrete’s structural integrity, causing spalling to occur and consequently resulting in a reduction in unit weight [3,32]. The significant increase in weight loss at higher temperature can be attributed to the escape of chemically bound water from the cement hydration products, which does not easily evaporate but is only lost after decomposition of the hydrates [31]. The weight loss of the concrete further soared with increments of DPF content at all elevated temperature levels, and this decrement was more pronounced with the escalation of the temperature. Relative to the control mix, the unit weight values of mixes D1-0, D2-0, and D3-0 were lower by 6.78%, 27.12%, and 62.17%, respectively, at a temperature of 200 °C. Similarly, when subjected to a temperature of 400 °C, the unit weight of mixes D1-0, D2-0, and D3-0 were reduced by 21.28%, 51.06%, and 88.30%, respectively, relative to the control. A comparable trend was found at both 600 °C and 800 °C. When the concrete was exposed to a temperature of 800 °C, the highest reduction in unit weight with the addition of DPF was noticed with reductions in unit weight by 54.59%, 76.06%, and 112.75% for mixes D1-0, D2-0, and D3-0, respectively, relative to the control. The reduction in unit weight with the addition of DPF when subjected to elevated temperatures can be ascribed to the fact that DPF entraps air during mixing and absorbs water; this, together with difficult packing and compaction, leads to higher porosity in the hardened concrete. The voids created by the DPF further facilitated dehydration or the evaporation of water from the cement matrix when subjected to higher temperature, hence resulting in a further reduction in unit weight. Additionally, the voids generated by the DPF created additional weak paths within the concrete microstructure where spalling could easily occur because of internal thermal stresses caused by the elevated temperature, which consequently reduces the unit weight. Furthermore, as DPF is an agricultural, organic, and fibrous material [13,33], when subjected to higher temperature it can easily degrade. When degraded, it creates additional weak paths for the easy dehydration of water and disintegration of the concrete, thus reducing the concrete’s unit weight.

Silica fume as SCM reduced the weight loss of the DPF concrete when exposed to elevated temperatures of up to 400 °C. At 200 °C, in comparison to the control, the weight loss of DPF concretes with 5% silica fume, namely D1-5 and D2-5, was less by 13.56% and 6.78%, respectively. Similarly, for DPF concretes with 10% silica fume, namely D1-10, D2-10, and D3-10 the weight loss was less by 45.76%, 27.12%, and 1.69%, respectively. For mixes containing 15% silica fume, only mix D1-15 had a lower weight reduction by 18.64% compared to the control. At 400 °C, mixes D1-5, D2-5, D1-10, D2-10, and D15-1 had lower weight losses of 9.57%, 3.19%, 10.64%, 13.83%, and 20.21%, respectively, compared to the control mix. Additionally, comparing the weight loss of mixes without any silica fume, i.e., mixes D1-0, D2-0, and D3-0 with their corresponding mixes containing 5%, 10%, and 15% silica fume, it can be observed that the latter mixes with silica fume have lower weight loss than their corresponding mixes without silica fume at 200 °C and 400 °C. This is to say that the addition of silica fume partially alleviated the undesirable effect of DPF addition on the weight loss and deterioration of the concrete when exposed to temperatures of up to 400 °C. The decline in weight loss caused by silica fume can be ascribed to the densification of the microstructure that is due to the pozzolanic reaction between the SiO_2_ from silica fume and the Ca(OH)_2_ from cement. This reduced the evaporation of water and the deterioration of the concrete. Another reason can be ascribed to the higher thermal resistance of silica fume when related to that of cement, as reported by some studies [34,35]; this might reduce the deterioration of the concrete’s microstructure because of resistance of higher heat. Similar findings have been reported by Sancak and Sari [36]. The incorporation of silica fume into the DPF-reinforced concrete increased the weight when subjected to temperatures of 600 °C and above. At 600 °C, compared to the control mix, for mixes containing 5% silica fume there is an escalation in weight loss by 54.6%, 74.7%, and 104.6% for D5-1, D5-2, and D5-3, respectively. Likewise, compared to the control mix, the weight loss for mixes D10-1, D10-2, and D10-3 were higher by 69.6%, 94.03%, and 114.8%, respectively. A similar results trend was observed at 800 °C, when the increment was more pronounced compared to at 600 °C. For instance, at 800 °C, there was an escalation in weight loss by 83.5%, 92%, and 129.3% for mixes D10-1, D10-2, and D1-3, respectively, relative to the control. The peak increment in weight loss was observed in mixes containing 15% silica fume when subjected to a temperature of 800 °C, where increments of 96.7%, 118.8%, and 134% were found for mixes D15-1, D15-2, and D15-3, respectively, compared to the control mix under the same temperature. The escalation in weight loss when silica fume was added was credited to the decomposition of C-S-H at higher temperature, as more C-S-H is formed in the concrete containing silica fume because of the pozzolanic reaction between the cement hydration products and the reactive silica (SiO_2_). This leads to increased porosity, microcracks, and deterioration and, hence, causes lower unit weight. Additionally, at higher temperatures above 350 °C, the Ca(OH)_2_ easily decomposes and converts to lime (CaO) by losing water. During cooling, the expansion of lime takes place, which easily causes an expansion in volume and damage resulting in increased weight loss [1,37]. Aydın and Baradan [37] have also reported similar observations in concrete containing silica fume subjected to high temperature.

### 3.2. Compressive Strength

One of the major effects of fire or high temperature on concrete is the reduction in its structural performance. The compressive strength is the main structural performance property of the concrete. The compressive strengths and relative strengths of the DPF-reinforced concrete after subjection to normal temperature (24 °C), 200 °C, 400 °C, 600 °C, and 800 °C are presented in Table 4. The compressive strengths of the concrete at any temperature decreased with increments in the percentage addition of DPF. For instance, at 200 °C, the compressive strengths of mixes D1-0, D2-0, and D3-0 were lower relative to the control by 2.54%, 13.46%, and 26.45%, respectively, while at 600 °C, the strengths of D1-0, D2-0, and D3-0 were lower by 31.1%, 34.19%, and 54.95%, respectively, related to that of the control. A similar results trend was seen at 400 °C and 800 °C. The reduction in strength was due to the fact that, when added to the concrete, DPF increased the pore volume. This, in addition to the higher ductile structure of the fiber compared to the aggregate and cement paste, caused extra discontinuities in the cementitious matrix. With load application, premature failure arose from the discontinuous path, therefore reducing the compressive strength [38]. Furthermore, the weak bonding between the DPF and cement matrix could also encourage reductions in strength at both lower and elevated temperatures [13]. Similar findings have been reported by Çavdar [38] for FRC made with different fibers, such as carbon fiber, glass fiber, and polyvinyl alcohol (PVA), when subjected to elevated temperatures. The compressive strength and relative strength of the DPF concrete mixes increased when subjected to elevated temperatures up to 400 °C, irrespective of the DPF and silica fume contents, as shown in Table 4. Considering the mixes without silica fume, in comparison to their respective compressive strengths at normal temperature, the control mixes had increased compressive strength by 3.07% and 15.92% at 200 °C and 400 °C, respectively, while the strength of mix D1-0 increased by 4.92% and 13.27% at 200 °C and 400 °C, respectively, and the strength of mix D2-0 increased by about 13.19% and 15.79% at 200 °C and 400 °C, respectively. Similar improvements in strength can be observed in other mixes containing different proportions of DPF and silica fume, where there is an improvement in compressive strength when subjected to elevated temperatures of up to 400 °C. Demirel and Keleştemur [1] reported an increase in compressive strength when they heated concrete containing pumice and silica fume as SCM to a temperature of up to 400 °C. Tanyildizi [12] also found increase in compressive strength when polypropylene-fiber-reinforced concrete (PPFRC) containing silica fume was heated to a temperature of up to 400 °C, while Poon and Azhar [8] found an increase in strength when subjecting concrete containing fly ash and slag as SCM to a temperature of 200 °C. The increase in strength might have been due to the hydrothermal reactions in the cement paste that led to the creation of excess tobermorite generated from the reaction between the calcium oxide in cement and the unhydrated silica (SiO_2_) from either the cement or the silica fume [1]. Another reason might have been a surge in the Van der Waals forces between the gel particles occurring from the evaporation of water by the high temperature [7,39].

The compressive strengths and relative strengths of the DPF-reinforced concrete reduced significantly when heated to temperatures of 600 °C and 800 °C irrespective of the DPF and silica fume contents. However, compared to the control mix, mixes with DPF only or with DPF and silica fume exhibited more significant reductions in strength at higher temperatures above 400 °C. For the control mix, there was a reduction in strength by 28.41% and 46.75% when heated to 600 °C and 800 °C, respectively, compared to its strength at normal temperature. For mixes with only DPF without silica fume, in comparison to their respective strengths at normal temperatures, when exposed to temperatures of 600 °C and 800 °C, mix D1-0 had reductions of 48.5% and 61.8%, respectively; for mix D2-0 there were reductions of 40.21% and 57%, respectively, and for D3-0 there were reductions of 57.8% and 68.7%, respectively. Tanyildizi [12] also observed reductions in compressive strength for PPFRC containing silica fume when subjected to elevated temperatures of more than 400 °C. The reduction in strength might have been due to DPF being an organic material easily degraded and disintegrated when subjected to very high temperatures. After disintegration, the DPF might leave pores, cause expansion, and weaken the aggregate–cement paste matrix. This causes microcracks to form because of the high-thermal internal stresses, hence resulting in a significant reduction in strength. Additionally, at a temperature above 270 °C, loss of chemically bound moisture from the C-S-H gel begins to take place. Further heating above this temperature causes decomposition of the hydrated C-S-H to continue and reach its peak at about a temperature of between 535 °C and 710 °C. This degradation process causes thermo-chemical spalling to occur, causing microcracks and weakening the structural integrity of the concrete, hence decreasing its strength [40]. Similar discussions were also reported by some researchers [40,41].

From Table 4, the compressive strengths and relative strengths of DPF mixes with different dosages of silica fume were higher compared to the corresponding DPF mixes without silica fume at temperatures of up to 600 °C for mixes containing up to 10% silica fume and temperatures of up to 400 °C for mixes with 15% silica fume. The further improvement in the compressive strength of the DPF-reinforced concrete at elevated temperatures that was due to silica fume addition might be attributed to the following: calcium hydroxide (Ca(OH)_2_) from cement hydration reacts when heated to higher temperatures, decomposing and converting to lime (CaO) and water vapor at about 350 °C. In the process of cooling, expansion of the CaO takes place and causes serious damage to the cement matrix, causing internal stresses and microcracks, consequently reducing the strength. Silica fume, because of its high SiO_2_, consumes the excess Ca(OH)_2_ and produces tobermorite and C-S-H, which can improve strength. This pozzolanic reaction hinders the conversion of Ca(OH)_2_ into lime and prevents the expansion effects [1]. However, the relative and compressive strengths of the DPF concrete mixes drops significantly with the increments in silica fume content when subjected to an elevated temperature of 800 °C, as seen in Table 4, where none of the DPF mixes containing any amount of silica fume were able to retain up to 40% of their compressive strength after subjection to a temperature of 800 °C, for instance, relative to the strength of the control. Similar findings have been reported by Behnood and Ziari [7] and Tanyildizi [12]. This significant reduction in strength can be ascribed to the fact that there is severe decomposition and deterioration of the C-S-H gels at 800 °C, which escalates the thermo-chemical spalling and microcrack formation, thereby leading to a substantial drop in strength [8,40].

### 3.3. Modeling Using Response Surface Methodology

#### 3.3.1. Response Surface Methodology

RSM is a special technique, one of the most suitable mathematical and statistical techniques for developing model equations to predict one or more responses (properties) using a single variable or a combination of several input variables. Furthermore, RSM has been found to be one of the most appropriate methods for performing optimization through the multi-objective optimization by setting up the desired goals for both the variables and responses [42,43]. The main packages available for RSM analysis are the design expert and Minitab software. In the RSM package, there are several model design types that can be selected for mix proportioning, modeling, and optimization. Some of the model design types include central composite, Box–Behnken, user defined, historical data, one factor, miscellaneous, etc. The choice of each mode design type depends on the number of inputs and their level of variations [43,44].

The relationship between the input variables and responses developed by the RSM can be represented by a generalized equation in the form of a linear relationship, as shown in Equation (3), when curvature does not exist. However, in actuality, curvature mostly exists, thus making the linear relationship not appropriate. Hence, polynomials with second or higher degrees, as given by Equation (4), are best suited to represent the RSM generalized equation [44,45].

(3) $$Y=r_{0}+r_{1}X_{1}+r_{2}X_{2}+\ldots \;rX_{n}+Є$$

(4) $$Y=r_{0}+\sum_{i=1}^{n}r_{i}\chi X_{i}+\sum_{i=1}^{n}r_{ii}X_{i}^{2}+\sum_{i<}\sum_{j}r_{ij}X_{i}X_{j}+Є$$

where *Y* denotes the output (response), *r*_0_ denotes the intercept where *X*_1_ = *X*_2_ = 0, *r*_1_ and *r*_2_ denote the coefficients of the first and second variables, respectively, *X*_1_ and *X*_2_ denote the first and second input (variables), respectively, *i* and *j* denote the linear and quadratic coded values, respectively, for the input, n denotes the number of inputs, and Є denotes the error.

RSM is one of the most suitable mathematical and statistical techniques for the mix proportioning of concrete, especially when many additives (variables) are used in the concrete. It is used to investigate the effects of one or more variables on the performance of the concrete. Additionally, RSM is best used for developing model equations for predicting one or more properties of the concrete, meaning the said property of the concrete can be calculated using the model equation without the need for carrying out experimental work or other field studies. RSM can also be used to optimize and achieve the best properties of the concrete using multi-objective optimization. This process will give the mix combinations that can be used to achieve the desired or highest properties of the concrete. Several studies have utilized RSM for mix proportioning, modeling, and optimization of different concrete properties using RSM [43,46,47,48].

For the RSM design, analysis, and optimization, Design Expert Version 11 software was utilized in this research. The Box–Behnken design was selected and adopted for the design because of its suitability. The variables used were DPF, silica fume, and temperature. All the variables were coded with low as −1, middle as 0, and high as +1. The levels and ranges for all the variables are presented in Table 5. The responses considered for the RSM analysis were weight loss, compressive strength, and relative compressive strength. The mixes were developed by the RSM and tested for the responses under consideration. The experimental results were used for developing the models and carrying out multi-objective optimization. The mix proportions and experimental results are presented in Table 6.

#### 3.3.2. Analysis of Variance

RSM was used to create models for forecasting the weight loss and strengths of DPF-reinforced concrete under severe conditions (subjected to elevated temperatures). The analysis of variance (ANOVA) was used to test the statistical significance and adequacy of the models. In all the models, D, S, and T represent DPF, silica fume, and temperatures, respectively. All the models and each of their terms were tested for statistical significance using a probability test, i.e., *p*-value. For a model or its term to be statistically significant, the probability of its Fisher statistical value (F-value) must be less than 0.05. In other words, if *p* < 0.05, then the model or its term is significant, meaning that the null hypothesis that there is no relationship between the variables and the models is false and, therefore, rejected. As shown in Table 7, models for estimating the weight loss, compressive strength, and relative strength were all significant as their *p*-values are less than 0.05 (based on confidence interval). With regards to the models’ terms, for weight loss, the D, T, D × T, and T^2^ were statistically significant in the model as they have *p* < 0.05, while the terms S, D × S, D^2^, and S^2^ were not significant statistically as their *p*-values were more than 0.05. Similarly, for the compressive strength model, the significant terms with *p* < 0.05 were D, S, T, S^2^, and T^2^, while the terms D × S, D × T, S × T, and D^2^ were not significant. Lastly, for the relative strength model, only the terms T and T^2^ have *p*-values less than 0.05 and are said to be significant, while all other model terms were not significant with P-values less than 0.05. The significance of each model relative to its lack of fit needs to be checked. Lack of fit is the measure of the predicted model that misses the observations. A nonsignificant lack of fit denotes a good model, i.e., the *p*-value of its lack of fit should be larger than 0.05. The weight loss, compressive strength, and relative strength models all have a nonsignificant lack of fit with respect to their pure errors, as the *p*-values of their lack of fits are greater than 0.05. Hence, the models have a good fit with their corresponding experimental data. The F-values of 3.58, 3.73, and 4.23 for weight loss, compressive strength, and relative strength models, respectively, indicated that there are 12.51%, 11.79%, and 9.85% probabilities, respectively, that a lack of fit of those values could occur because of distorted data (noise) [43,49]. Therefore, the developed models given in Equations (5), (6), and (7) for weight loss, compressive strength, and relative strength, respectively, can be used for predicting the properties of DPF-reinforced concrete containing silica fume under severe environmental conditions (high temperatures).

(5) $$W_{L}\left(\%\right)=7.88-1.182\times D-0.226\times S-0.042\times T+0.016\times D\times S+0.0017\times D\times T+0.00018\times S\times T+0.195\times D^{2}\;+0.0075\times S^{2}+0.000065\times T^{2}$$

(6) $$F_{c}=4.205+9.266\times D+4.648\times S-0.226\times T-0.163\times D\times S+0.0041\times D\times T+0.0016\times S\times T-3.881\times D^{2}\;-0.30\times S^{2}-0.00039\times T^{2}$$

(7) $$R_{c}=19.5-11.16\times D-2.082\times S+0.704\times T-0.875\times D\times S-0.021\times D\times T+0.0046\times S\times T+6.828\times D^{2}\;+0.372\times S^{2}-0.00097\times T^{2}$$

where *W_L_* stands for weight loss in %, *F_c_* stands for compressive strength in MPa, *R_c_* stands for residual strengths in %, *D* and *S* represent DPF and silica, respectively, in %, and *T* represents elevated temperature in °C.

The predicted residual error sum of squares (PRESS) was used to further check the correlation and efficiency of the models statistically. The coefficient of determination (R^2^) was calculated using PRESS and the results are summarized in Table 8. R^2^ values range between zero (0) and one (1), with models having higher R^2^ values close to one said to be highly correlated with the experimental data and have a very good predicting ability. On the other hand, models with lower R^2^ values are said to have a poor fitness accuracy with the experimental data and low predicting ability. The models developed to forecast the weight loss, compressive strength, and residual strength all have a very high R^2^ values larger than 0.94. The R^2^ values of 0.998, 0.945, and 0.946 for weight loss, compressive strength, and residual strength models, respectively, denote that the models have very high fitness. Only about 2%, 5.5%, and 5.6% of the experimental results for weight loss, compressive strength, and residual strength models, respectively, cannot be fully correlated into the models. The variability of predicted and adjusted R^2^ numbers for each of the models was further used to validate its fitness and adequacy. For a well-fitted model, the variation of its predicted and adjusted R^2^ values should be lower than 0.2. If not, the models and/or the experimental data are said to have problems because of large block effects. If this problem occurs for the models to be used, necessary corrections are required by carrying out any of the following: model reduction, looking for outliers, transformation, or using higher-order polynomials for the model [44]. From Table 8, the variation of the predicted and adjusted R^2^ values for the weight loss model is less than 0.2; therefore, the model and experimental data said to have no problem and the developed model equation can be used even without removing the nonsignificant terms. However, for the compressive and relative strength models, their adjusted R^2^ minus predicted R^2^ numbers are higher than 0.2; therefore, something is wrong with the model and/or the experimental data. To solve this problem, model reduction was carried out using a backward elimination method by which the nonsignificant model terms were removed/reduced. Following backward elimination, the models’ predicted and adjusted R^2^ values became reasonable in compliance as their variation was reduced to less than 0.2. Therefore, for compressive strength and relative strength models, Equations (9) and (10) are appropriate for predicting their values. Moreover, the consistencies of the models were checked by comparing their respective standard deviations to their mean values. All the models have a reasonably lower standard deviation when compared to their means, and, hence, this justifies the less variability between the models and their experimental data. The models’ high adequate precision values greater than 4 also justified the models being appropriate for navigating the design space [43,50].

The developed models after model reduction are given as Equation (8), Equation (9), and Equation (10) for weight loss, compressive strength, and relative strength, respectively.

(8) $$W_{L}\left(\%\right)=5.843-0.241\times D-0.041\times T+0.00166\times D\times T+0.000066\times T^{2}$$

(9) $$F_{c}=10.212-6.264\times D+5.111\times S+0.254\times T-0.308\times S^{2}-0.000399\times T^{2}$$

(10) $$R_{c}=16.45+1.78\times S+0.598\times T-0.000943\times T^{2}$$

#### 3.3.3. Diagnostic Plots

**Figure 4:**
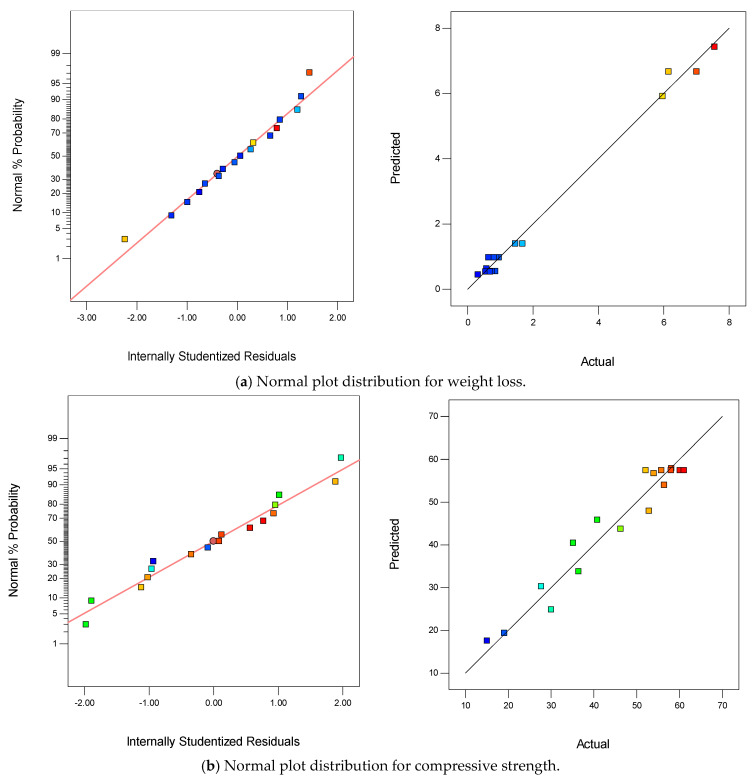

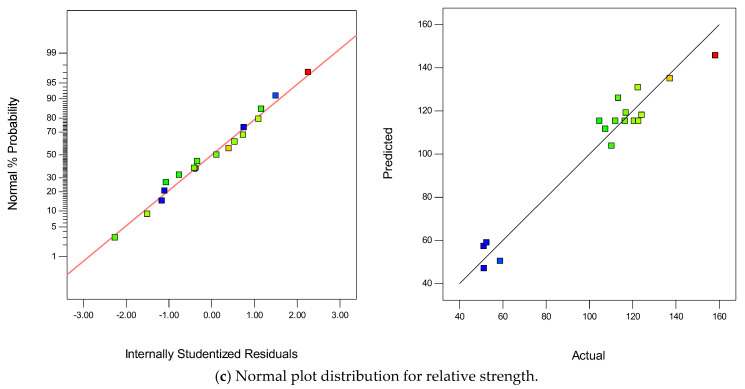
Normal plot distributions for (**a**) weight loss model, (**b**) compressive strength model, and (**c**) relative strength model.

The normal plot against studentized residuals and predicted versus actual plots for all the responses are presented in Figure 4a–c. These diagnostic plots were used to check and validate the normality of the residuals and the correction between the models and experimental data graphically. From the figures displayed for the normal plots for all the models, the plotted data points were closely aligned across the straight normal lines. Therefore, all the models were said to follow the normal probability distribution function. The weight loss model has the best fit in terms of the normal plots; this justified its highest R^2^ value among the models and, hence, the best predicting ability. With regards to the predicted versus actual plots, for all the models the experimental data were closely corrected with the predicted models as the data points fall along the reference straight trend line. Here also, the model for weight loss has the best fitness, which justified its high degree of correlation and predictability.

**Figure 5:**
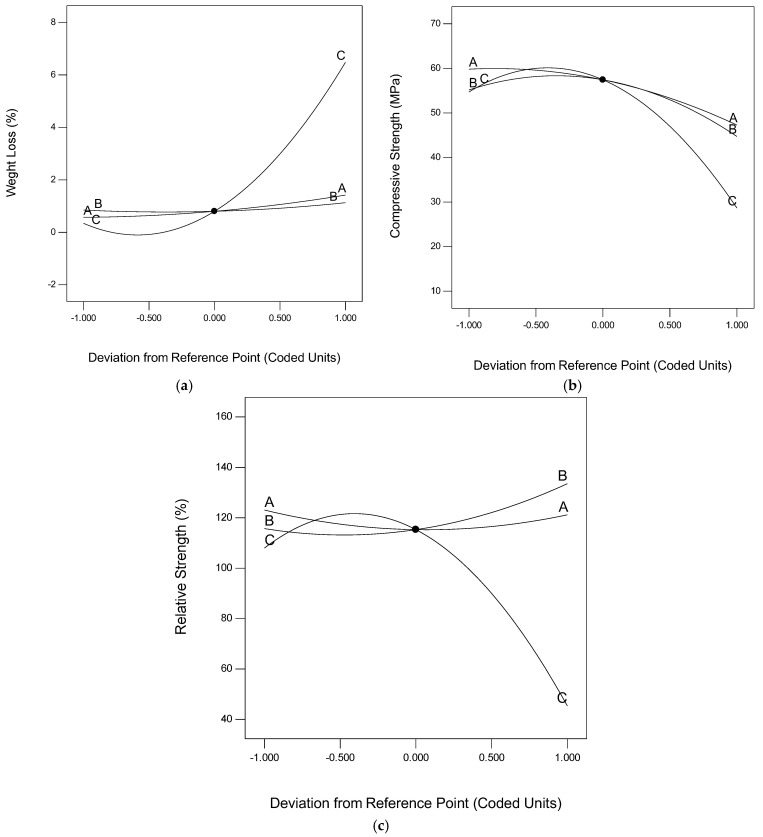
Perturbation plots. (**a**) Weight loss (%); (**b**) compressive strength (MPa); (**c**) relative strength (%).

The perturbation plots were used to investigate and compare the effects of all the variables at a particular reference point on a response. This is normally measured by how each of the variables moves away from the reference point (taken as the zero point on the plot), while all other variables are held constant at that point [43,50]. In the perturbation plots shown in Figure 5, the letters A, B, and C represent DPF, silica fume, and temperature, respectively. In all the figures, the plot for temperature (i.e., C) has the steepest curvature; this implies that temperature is the most sensitive variable in terms of weight loss, compressive strength, and relative strength based on the experimental results. The positive curvature of C in Figure 5a means that temperature is increasing the weight loss of the DPF-reinforced concrete, while the negative curvatures of C in Figure 5b and Figure 5c imply that the temperature declined the compressive and relative strengths, respectively. From Figure 5a, silica (B) has the most relatively flat curvature, which showed that it has the least sensitivity in terms of weight loss. From Figure 5b,c, by careful observation, the curvature of A is the flattest; therefore, DPF has the least sensitivity in terms of compressive strength and relative strengths based on the experimental results.

#### 3.3.4. Response Surface Plots

**Figure 6:**
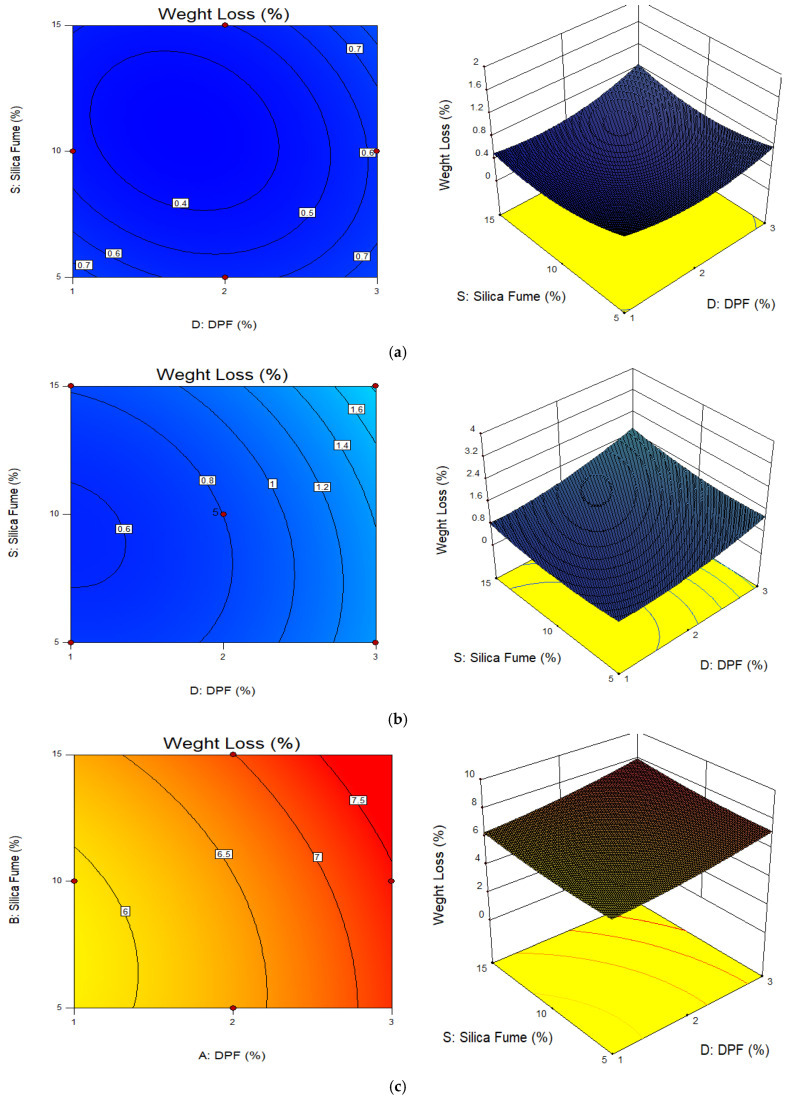
2D contour and 3D response surface plots for weight loss. (**a**) Temperature 200 °C; (**b**) temperature 400 °C; (**c**) temperature 600 °C.

The 2D contour and 3D response graphs were plotted to assess the interactive relationship between the variables and responses under consideration. Figure 6 presents the 2D and 3D plots for weight loss, where the plots were drawn as a function of the two variables (DPF and silica fume) and the third variable (temperature) was held constant. Figure 6a, Figure 6b, and Figure 6c are the plots for the weight loss at temperatures of 200 °C, 400 °C, and 600 °C, respectively. The elliptical contour curve shows a perfect interactions between the variables (DPF and silica fume) on the response (weight loss) [51,52]. However, by careful observation, the interactions between the variables reduced as the temperature increased, i.e., the contour ellipticity of the contours tends to reduce, the contour curves becoming straight. This might be due to the effect of high temperature, which degrades the properties performance of the variables. Additionally, the 3D plots showed that the weight loss increased with increments in elevated temperature, DPF, and silica fume addition. However, at 200 °C, the effect of DPF and silica fume on the weight loss is minimal, as shown in Figure 6a.

**Figure 7:**
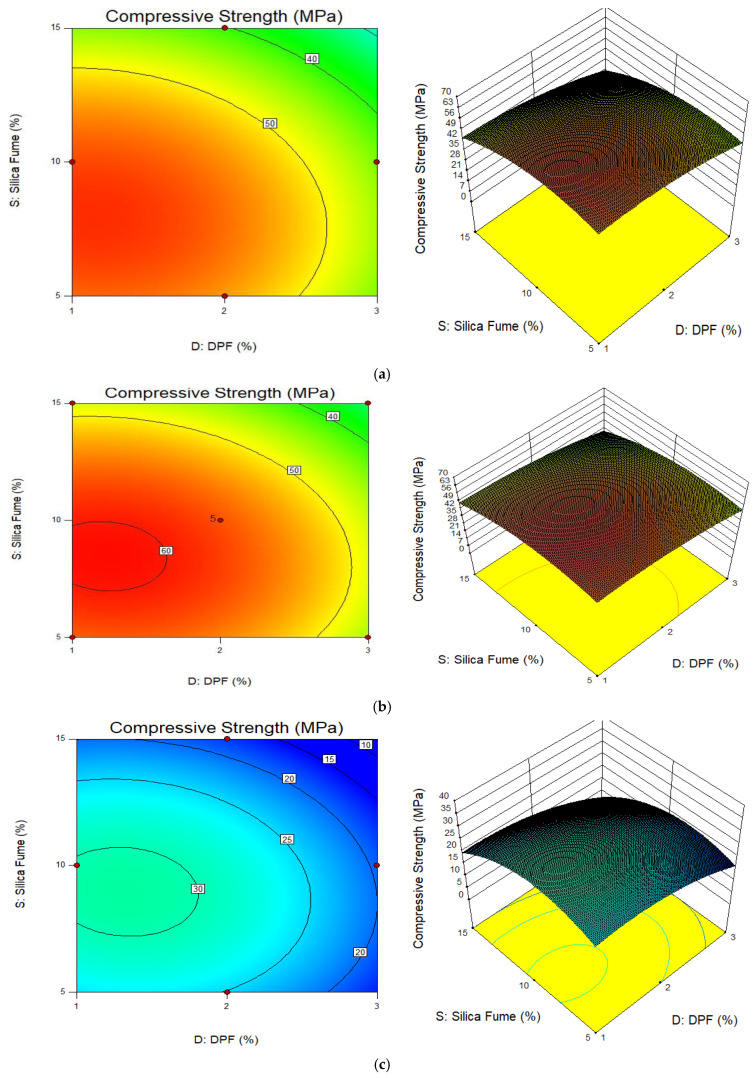
2D contour and 3D response surface plots for compressive strength. (**a**) Temperature 200 °C; (**b**) temperature 400 °C; (**c**) temperature 600 °C.

**Figure 8:**
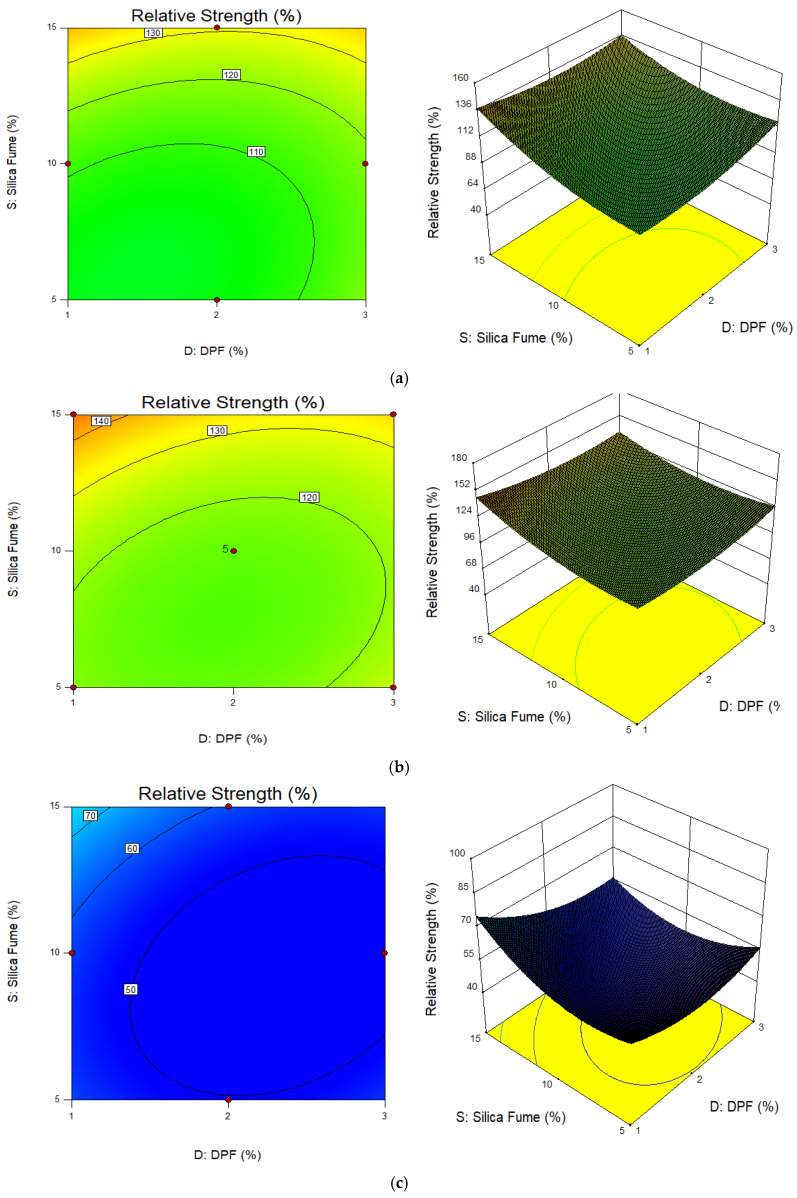
2D contour and 3D response surface plots for relative strength. (**a**) Temperature 200 °C; (**b**) temperature 400 °C; (**c**) temperature 600 °C.

Figure 7 and Figure 8 present the plots (2D contour and 3D response surface) for compressive strength and relative strength responses, respectively, under different elevated temperatures. The contour plots for both compressive and relative strengths at all temperatures are elliptical in shape. This reveals that there is a perfect interaction between the variables, i.e., DPF and silica fume, on the compressive strength and relative strength responses. The ellipticity of the contours tends to be the same under all temperature conditions; hence, it is assumed that the interactions between DPF and silica fume do not have much significant effect on the weight loss of the concrete at temperatures up to 800 °C. The 3D plot relationships show that with reference to temperature, both the compressive strength and relative strength responses increased when heated to between 200 °C and 400 °C, while at 600 °C there was significant reduction in both compressive and relative strengths. Furthermore, from the 3D plots, it is shown that the compressive and relative strengths were enhanced with the replacement of cement by silica fume and reduced with an increase in the percentage of additional DPF.

#### 3.3.5. Multi−Objective Optimization

One of the main advantages of using RSM is the ability to carry out mix optimization by setting out some goals to achieve the desired output. In this study, optimization was carried out to achieve the optimum performance of the DPF-reinforced concrete in terms of strength and weight retention after subjection to severe environmental conditions (elevated temperatures). The summary of the optimization goals and criteria is presented in Table 9, where the compressive strengths and relative strengths were maximized and weight loss after subjection to high temperature was minimized. The variables, i.e., DPF, silica fume, and temperature, were kept within the ranges used in the study. From the results of optimization, five possible solutions were obtained, as shown in Table 9, and they were presented based on desirability. The optimum responses were achieved by adding 1% DPF by weight of binder material, replacing 12.1% of cement with silica fume, and heating the samples at 317 °C for 2 h. The desirability of the best solution was 91.3%.

#### 3.3.6. Model Validation

The developed models for predicting the weight loss, compressive strength, and residual strength of the DPF-reinforced concrete under high temperature were validated experimentally by carrying out a series of additional experiments and comparing the results with those calculated using the developed model equations. This model was carried out to check the practical ability of the proposed models. The variables from the optimization solutions and some randomly selected proportions of the variables were used as the mix proportions for preparing the samples for the experimental work, while other constituent materials were kept constant. The responses were also estimated from the models using the same proportions of variables as those used for the experiments. The percentage errors between the experimental and predicted models were then calculated using Equation (11):

(11) $$\in =\frac{M-N}{M}\times 100$$

where ∈ represents the percentage error, and *M* and *N* are the experimental and predicted (model) responses, respectively.

Table 10 summarizes the results of the models’ validations. Based on the experimental and predicted results, the models have average errors of less than 6%. The weight loss has an error of 5.72%, the compressive strength model has an average error of 5.16%, and the relative strength model has an average error of 5.04%. Therefore, the established models for forecasting the weight loss and strengths of DPF-reinforced concrete under elevated temperature are said to be highly correlated with a very good degree of predictability.

## 4. Conclusions

In this study, the effect of elevated temperature on the strengths of DPF-reinforced concrete containing silica fume as a partial substitute for cement was investigated. Based on the experimental and RSM analyses, the following conclusions are summarized:

(1) The weight loss of the DPF-reinforced concrete worsened with an increase in temperature and DPF addition. The weight loss escalated significantly when subjected to temperatures of 600 °C and above.
(2) The compressive and relative strengths of the DPF-reinforced concrete was enhanced when exposed to temperatures of up to 400 °C, irrespective of the DPF and silica fume contents. However, at higher temperatures of above 400 °C, there was significant loss in strength in the concrete.
(3) The compressive strength and relative strength of the DPF-reinforced concrete decreased with increments in DPF content, irrespective of the temperatures it was subjected to.
(4) Silica fume as a partial substitute for cement in DPF-reinforced concrete improves its heat resistance when subjected to a temperature up to 400 °C, where the weight loss declined and the strengths improved with the replacement of up to 10% cement with silica fume, irrespective of the DPF contents in the concrete. However, at temperatures of 600 °C and above, the silica fume addition significantly decreased the strength of the DPF-reinforced concrete.
(5) Models were developed to predict the weight loss and compressive strengths of DPF-reinforced concrete in severe conditions (elevated temperatures) using RSM. The developed models have a very high degree of correlation and predictability. The models were said to have an average error of less than 6% when validated experimentally.
(6) Based on the multi-objective optimization, the highest performance of the DPF-reinforced concrete under elevated temperature was achieved by adding 1% DPF by weight of binder materials, replacing 12.14% of the cement using silica fume, and subjecting the concrete to a temperature of 317 °C. The optimization result has a very high desirability of 91.3%.
(7) Therefore, for DPF-reinforced concrete to be used in areas subjected to high temperature, it is recommended to use silica fume as a partial replacement for cement in the mix to improve its heat resistance.

## Figures and Tables

**Table 1:** Chemical properties of cementitious material.

Oxides	Compositions (%)	
	Cement	Silica Fume
Al_2_O_3_	5.390	0.260
CaO	65.180	0.210
Fe_2_O_3_	3.400	0.050
SiO_2_	19.710	95.850
MgO	0.910	0.450
Na_2_O	0.170	−
K_2_O	1.22	−
TiO_2_	0.24	−
SO_3_	3.51	1.00
P_2_O_5_	0.09	−
MnO	0.18	−
LOI	2.38	2.80

**Table 2:** Physical and mechanical characteristics of DPF.

Characteristics	Values
Diameter	0.20–1.00 (mm)
Length	20.00–30.00 (mm)
Bulk density	877.43 ± 4.80 (kg/m^3^)
Moisture content	10.20 ± 0.40 (%)
Water absorption to saturation	102.65 ± 3.30 (%)
Tensile strength	203.24 ± 30 (MPa)
Elongation at break	13.50 ± 20 (%)
Modulus of elasticity	3.35 ± 1.50 (GPa)

**Table 3:** Mix proportions and constituent materials.

Mix	Variable (%)		Quantity (kg/m^3^)						
	DPF (%)	Silica Fume (%)	Cement	Silica Fume	Fiber	Fine Aggregate	Coarse Aggregate	Water	S. P
Control	0	0	490	0	0	750	905	185	4.9
D1-0	1	0	490	0	4.9	750	905	185	4.9
D2-0	2	0	490	0	9.8	750	905	185	4.9
D3-0	3	0	490	0	14.7	750	905	185	4.9
D1-5	1	5	465.5	17.9	4.83	750	905	185	4.8
D2-5	2	5	465.5	17.9	9.67	750	905	185	4.8
D3-5	3	5	465.5	17.9	14.50	750	905	185	4.8
D1-10	1	10	441	35.8	4.77	750	905	185	4.8
D2-10	2	10	441	35.8	9.54	750	905	185	4.8
D3-10	3	10	441	35.8	14.30	750	905	185	4.8
D1-15	1	15	416.5	53.7	4.70	750	905	185	4.7
D2-15	2	15	416.5	53.7	9.40	750	905	185	4.7
D3-15	3	15	416.5	53.7	14.11	750	905	185	4.7

**Table 4:** Compressive strength results after subjection to elevated temperatures.

Mixes	Compressive Strength (MPa)					Relative Compressive Strength (%)			
	24 °C	200 °C	400 °C	600 °C	800 °C	200 °C	400 °C	600 °C	800 °C
Control	43.91	45.26	50.90	31.44	23.38	103.1	115.9	71.6	53.2
D1-0	42.04	44.11	47.62	21.66	16.08	104.9	113.3	51.5	38.2
D2-0	34.61	39.17	40.07	20.69	14.88	113.2	115.8	59.8	43.0
D3-0	35.02	33.29	20.22	14.79	10.95	95.1	57.7	42.2	31.3
D1-5	46.22	49.24	54.03	26.39	17.96	106.5	116.9	57.1	38.9
D2-5	51.17	56.45	59.11	30.07	13.17	110.3	115.5	58.8	25.7
D3-5	36.00	43.96	40.83	21.51	10.11	122.1	113.4	59.8	28.1
D1-10	54.12	58.12	66.69	27.72	14.15	107.4	123.2	51.2	26.1
D2-10	49.80	54.98	57.43	21.47	12.91	110.4	120.7	43.1	25.9
D3-10	37.25	46.27	36.50	19.12	10.54	124.2	98.0	51.3	28.3
D1-15	33.42	47.01	52.92	14.18	8.41	140.6	158.3	42.4	25.2
D2-15	28.74	35.20	45.52	15.08	7.34	122.5	158.4	52.5	25.5
D3-15	26.56	33.97	36.46	14.02	8.91	127.9	137.3	52.8	33.5

**Table 5:** Variables’ range and levels.

Variable	Symbol	Unit	Range and Levels		
			Low (−1)	Middle (0)	High (+1)
DPF	D	%	1	2	3
Silica fume	F	%	5	10	15
Temperature	T	°C	200	400	600

**Table 6:** Mix proportions and results from RSM.

Run	Factors			Responses		
	DPF (%)	Silica Fume (%)	Temperature (°C)	Weight Loss (%)	Compressive Strength (MPa)	Relative Strength (%)
1	2	10	400	0.81	61.08	122.7
2	1	10	600	5.97	27.72	51.2
3	2	15	200	0.69	35.2	122.5
4	2	10	400	0.88	52.14	104.7
5	1	10	200	0.32	58.12	107.4
6	2	10	400	0.64	58.02	116.5
7	1	5	400	0.85	54.03	116.9
8	2	5	200	0.55	56.45	110.3
9	3	15	400	1.68	36.46	137.3
10	2	5	600	6.15	30.07	58.8
11	3	10	600	7.56	19.12	51.3
12	2	15	600	7.01	15.08	52.5
13	2	10	400	0.72	55.8	112
14	2	10	400	0.96	60.11	120.7
15	3	5	400	1.46	40.83	113.4
16	3	10	200	0.58	46.27	124.2
17	1	15	400	0.75	52.92	158.3

**Table 7:** ANOVA summary.

Responses	Source	Sum of Squares	Mean Square	F Value	*p*-Value Prob > F	Significance
Weight Loss (%)	Model	107.18	11.91	353.38	<0.0001	significant
	D-DPF	1.44	1.44	42.63	0.0003	
	S-Silica Fume	0.16	0.16	4.65	0.0679	
	T-Temperature	75.34	75.34	2235.50	<0.0001	
	D × S	0.026	0.026	0.76	0.4123	
	D × T	0.44	0.44	13.12	0.0085	
	S × T	0.13	0.13	3.85	0.0907	
	D^2^	0.16	0.16	4.76	0.0654	
	S^2^	0.15	0.15	4.40	0.0740	
	T^2^	28.69	28.69	851.26	<0.0001	
	Lack of Fit	0.17	0.057	3.58	0.1251	not significant
Compressive Strength (MPa)	Model	3348.52	372.06	13.31	0.0013	significant
	D-DPF	313.88	313.88	11.23	0.0122	
	S-Silica Fume	217.57	217.57	7.78	0.0269	
	T-Temperature	1353.30	1353.30	48.42	0.0002	
	D × S	2.66	2.66	0.095	0.7668	
	D × T	2.64	2.64	0.094	0.7675	
	S × T	9.80	9.80	0.35	0.5724	
	D^2^	63.43	63.43	2.27	0.1757	
	S^2^	236.13	236.13	8.45	0.0228	
	T^2^	1043.31	1043.31	37.33	0.0005	
	Lack of Fit	144.14	48.05	3.73	0.1179	not significant
Relative Compressive Strength (%)	Model	15342.82	1704.76	13.70	0.0012	significant
	D-DPF	7.22	7.22	0.058	0.8166	
	S-Silica Fume	633.68	633.68	5.09	0.0586	
	T-Temperature	7850.04	7850.04	63.08	<0.0001	
	D × S	76.56	76.56	0.62	0.4585	
	D × T	69.72	69.72	0.56	0.4785	
	S × T	85.56	85.56	0.69	0.4344	
	D^2^	196.27	196.27	1.58	0.2495	
	S^2^	366.33	366.33	2.94	0.1299	
	T^2^	6280.83	6280.83	50.47	0.0002	
	Lack of Fit	662.51	220.84	4.23	0.0985	not significant

**Table 8:** ANOVA models validation.

Factors	Weight Loss (%)		Compressive Strength (MPa)		Relative Strength (%)	
	Before Reduction	After Reduction	Before Reduction	After Reduction	Before Reduction	After Reduction
Std. Dev.	0.18	0.27	5.29	4.99	11.16	11.44
Mean	2.21	2.21	44.67	44.67	104.75	104.75
C.V. %	8.30	12.21	11.83	11.18	10.65	10.93
PRESS	2.85	1.63	2386.7	745.3	10,926.1	2985.9
R^2^	0.998	0.992	0.945	0.923	0.946	0.895
Adjusted R^2^	0.995	0.989	0.874	0.888	0.877	0.871
Predicted R^2^	0.974	0.985	0.327	0.791	0.326	0.816
Adequate Precision	49.61	47.73	9.95	15.39	11.53	15.65

**Table 9:** Multi-objective optimization criteria.

Variables and Response	Goal	Lower Limit	Upper Limit	Solutions		
				Soln 1	Soln 2	Soln 3
D: DPF (%)	In range	1	3	1.0	1.0	1.14
S: Silica Fume (%)	In range	5	15	12.14	12.1	11.74
T: Temperature (°C)	In range	200	600	317	304	312
Weight Loss (%)	Minimize	0.32	7.56	0.133	0.16	0.11
Compressive Strength (MPa)	Maximize	15.08	61.08	61.08	61.08	61.08
Relative Strength (MPa)	Maximize	51.2	158.3	132.8	132.6	132.1
Desirability (%)				91.3	91.3	91.1

**Table 10:** Models’ experimental validations.

Response	Variables			Experimental	Predicted	Error (%)	Average Error (%)
	DPF (%)	Silica Fume (%)	Temperature (°C)				
Weight loss (%)	1.0	12.14	317	0.252	0.237	6.15	5.72
	1.14	11.74	312	0.221	0.209	5.61	
	2.0	10	400	0.799	0.849	6.26	
	1.5	7.5	300	0.14	0.132	6.07	
	2.5	12.5	500	3.535	3.316	6.21	
	3.0	15	600	7.571	7.268	4.00	
Compressive strength (MPa)	1.0	12.14	317	58.52	61.02	4.28	5.16
	1.14	11.74	312	56.83	61.03	7.39	
	2.0	10	400	58.2	55.75	4.20	
	1.5	7.5	300	59.16	62.11	4.99	
	2.5	12.5	500	35.33	37.56	6.32	
	3.0	15	600	7.27	7.55	3.78	
Relative strength (%)	1.0	12.14	317	127.62	132.86	4.11	5.04
	1.14	11.74	312	125.97	132.13	4.89	
	2.0	10	400	127.71	122.57	4.02	
	1.5	7.5	300	118.64	124.33	4.80	
	2.5	12.5	500	96.23	101.95	5.94	
	3.0	15	600	58.67	62.47	6.48	
